# Preferences for Electronic Modes of Communication Among Older Primary Care Patients: Cross-sectional Survey

**DOI:** 10.2196/40709

**Published:** 2023-05-24

**Authors:** Ilona Fridman, Ahmaya Smalls, Patrice Fleming, Jennifer Elston Lafata

**Affiliations:** 1 Cancer Care Quality Program Lineberger Cancer Center University of North Carolina Chapel Hill, NC United States; 2 Eshelman School of Pharmacy University of North Carolina Chapel Hill, NC United States; 3 Center for Equity in Research Duke Clinical and Translational Science Institute Duke University Durham, NC United States; 4 Lineberger Comprehensive Cancer Center Eshelman School of Pharmacy University of North Carolina Chapel Hill, NC United States

**Keywords:** electronic communication, patient preferences, digital health, patient portals, physician-patient communication, communication, text, phone, test, treatment, clinical, vaccination, survey, intervention, screening, social media, information

## Abstract

**Background:**

Health information delivered via daily modes of communication such as email, text, or telephone reportedly supports improved health behavior and outcomes. While different modes of communication beyond clinical visits have proven successful for patient outcomes, preferences for communication modes have not been comprehensively studied among older primary care patients. We addressed this gap by assessing patient preferences for receiving cancer screening and other information from their doctors’ offices.

**Objective:**

We explored stated preferences by communication modes through the lens of social determinants of health (SDOH) to gauge acceptability and equity implications for future interventions.

**Methods:**

A cross-sectional survey was mailed to primary care patients aged 45-75 years, in 2020-2021, which assessed respondents' use of telephones, computers, or tablets in daily life and their preferred modes of communication for different types of health information, including educational materials about cancer screening, tips for taking prescription medication, and protection from respiratory diseases from their doctors’ offices. Respondents indicated their willingness to receive messages from their doctors’ offices via each of the provided modes of communication, including telephone, text, email, patient portals, websites, and social media, on a 5-point Likert scale ranging from “unwilling” to “willing.” We present the percentage of respondents who indicated that they were “willing” to receive information via specific electronic mode. Chi-square tests were used to compare participants’ willingness by social characteristics.

**Results:**

In total, 133 people completed the survey (response rate 27%). The average respondent age was 64 years, 82 (63%) respondents were female, 106 (83%) were White, 20 (16%) were Black, and 1 (1%) was Asian. In total, 75 (58%) respondents had a bachelor’s degree or higher; 26 (20%) resided in rural areas, 37 (29%) in suburban areas, 50 (39%) in a town, and 15 (12%) in a city. The majority, 73 (57%), reported being comfortable with their income. Preferences of respondents for electronic communication about cancer screening were distributed as follows: 100 (75%) respondents were willing to receive information from their doctor’s office via their patient portal, 98 (74%) via email, 75 (56%) via text, 60 (45%) via the hospital website, 50 (38%) via telephone, and 14 (11%) via social media. About 6 (5%) respondents were unwilling to receive any communication via electronic modes. Preferences were distributed similarly for other types of information. Respondents reporting lesser income and education consistently preferred receiving telephone calls relative to other communication modes.

**Conclusions:**

To optimize health communication and reach a socioeconomically diverse population, telephone calls should be added to electronic communication, especially for people with less income and education. Further research needs to identify the underlying reasons for the observed differences and how best to ensure that socioeconomically diverse groups of older adults can access reliable health information and health care services.

## Introduction

According to a 2022 cross-national survey, people frequently use electronic devices to send or receive information from health care providers: about 41% of the US population accessed patient medical records [1] and 37% interacted with their providers using SMS text messages in the last 12 months [2]. At least in some health care settings, patients use electronic health communications with a higher frequency. For example, within 1 health system, 60% of patients used messaging through medical records at least once a year [3].

Complex health issues and limited time during clinical visits often require patients and providers to communicate outside of office visits. Providers today have an opportunity to send health information using multiple modes of communication including email, patient portals, telephone calls, or SMS text messaging. Electronic communication has been shown to have a positive influence on patients' outcomes, such as facilitating adherence to recommended cancer screening tests [4], prescribed treatments [5,6], and vaccinations [7].

While the majority of patients reported preferring in-person visits over electronic communication [8], patient preferences vary. For instance, patients chose electronic communication for follow-up visits if they needed to address a small health problem [9] or if they needed to learn their test results [10,11]. Past work has illustrated that patients are generally open to electronic communications that provide educational materials and increase their knowledge about their health. For instance, patients endorsed electronic communications for receiving additional explanations about their surgery [12], reminders about sunscreen [13], or immunization [14]. Patients reported that electronic health communication made them feel informed and connected with their health care providers [15]. To explore patients' preferences in detail, we focused our study on electronic communications related to health education, specifically on delivering information related to cancer screening, prescription medication, and protection against respiratory diseases including COVID-19. The goal of our work is to identify, which electronic modes of communication health care providers can use to best reach patients equitably.

While studies have been conducted to compare patients’ perceptions of different electronic modes [12,16], to date, there is no study that comparatively outlines the preferences of older primary care patients for electronic modes of communication. With age, people become eligible for evidence-based cancer screening, prone to chronic conditions that often require medications, and have an increased risk of complications from respiratory disorders, and therefore, need not only to use health care services [17] more often than younger patients but also to receive information about those services. At the same time, older patients tend to have low electronic literacy [18,19] and are less likely to use electronic communication than younger patients [20]. The rapid adoption of electronic communication by health care delivery organizations may create barriers for older patients when they need to access care [21]. We aimed to provide empirical data to inform the recommendations for communication modes, and thus improve access to health information and care.

We applied the social determinants of health (SDOH) framework to provide a detailed overview of preferences among patient populations that have historically faced challenges in accessing health information and health care. SDOH included conditions that frequently underline inequalities in health access and outcomes [22]. The most influential factors include economic stability, education, neighborhood, and social context [23]. On one hand, adopting electronic communications with patients could help mitigate inequalities. For instance, a systematic review showed that interventions that included electronic technologies helped to disseminate cancer-related information and significantly improve knowledge and outcomes among African American, Hispanic, and patients living in rural areas [24]. On the other hand, electronic communications, when not properly tailored to patient needs, could widen the divide benefiting only those who already have well-established access to health care [25,26]. For instance, a different study showed that African American patients and patients living in rural areas use the internet less than White patients and patients living in urban areas [27]. Thus, reaching patients via electronic modes that require an internet connection structurally puts those populations in a disadvantageous position. Consistent with the domains in SDOH, patients' age, gender, and socioeconomic status have also been found to be negatively associated with patients' engagement with electronic health information [28].

To prevent the negative influence of the rapid adoption of electronic communications, several solutions were proposed [28]. A critical step among them is assessing the preferences of people who have been historically marginalized and using results to develop interventions that are delivered to the populations via their preferred modes of electronic communication [29]. Following these recommendations, we focus our study on exploring patients' preferences for electronic modes of communication and comparing preferences among populations. Our overarching goal is to summarize patient preferences and differences in preferences among subgroups of patients defined by key SDOH to provide recommendations for which electronic modes of communication practitioners, researchers, and others to adopt when trying to reach patients living in different SDOH contexts.

## Methods

### Overview

We conducted a cross-sectional study among adult primary care patients. Patients were identified via the electronic health record of a large North Carolina–based health system. Eligible patients were those aged 45-75 years with a visit to any of the health system’s primary care clinics between July 1 and December 31, 2019. Among study-eligible patients, we randomly selected 500 participants and invited them to complete a mailed survey. The decision to use mail was intentional to facilitate the recruitment of a balanced sample and consider individuals with various levels of electronic literacy and access to electronic devices. The survey distribution via mail allowed people with different access to electronic devices an opportunity to be in the study. In a mailed introductory letter, we offered potential participants the option to complete the survey in paper-pencil format or online format.

Survey data collection was conducted in 2 waves. The first half of the surveys was sent in October 2020 and the other half was sent in January 2021. Potential participants received a mailed packet that included an introductory letter or information sheet, a paper-pencil version of the survey, and a refrigerator magnet of a local university. In the mailed introductory letter, we offered potential participants the option to complete the survey in paper-pencil format or online. They were invited to keep the magnet, regardless of their study participation (ie, survey completion). Respondents who chose to complete the paper-pencil survey were asked to mail it back to the study team in a prepaid envelope. A reminder packet was sent approximately 2-3 weeks later to the potential participants for which we received no response. The reminder packet also included a URL address, enabling responders to complete the survey online.

### Ethics Approval

The institutional review board of University of North Carolina at Chapel Hill, Office of Human Research Ethics approved the research (20-1168), exempting respondents from written informed consent due to low risk. The collected data were deidentified and stored on a shared drive protected by the IT services of the University.

### Measures

#### Overview

Survey items were developed for this study and are provided in Multimedia Appendix 1. The items were informed by our previous research that studied patient preferences [29,30] and patient-centered communication [31]. Those developing the survey had expertise in health communication, measure development, and health services research.

#### Access to Electronic Devices and Use of Electronic Communication Modes

Respondents first reported whether they had access to a computer, tablet, smartphone, basic cell telephone, and telephone landline, by choosing “yes” or “no” next to each option. They could choose more than 1 option or an option that stated, “no access to any of the above devices.” Respondents next indicated how frequently they communicated via SMS text messaging; used the internet (via a computer, tablet, or cell phone); and accessed social media such as Facebook, Twitter, Instagram, and YouTube. They reported their experiences using a Likert scale response that ranged from “Never” to “Every day.” Respondents were presented with a list of 6 electronic modes of communication (telephone calls, SMS text messages, emails, patient portals, hospital website, and social media). For each item, respondents were asked to indicate (yes or no) if they currently received information from their health care provider using each mode of communication.

#### Preferences for Modes of Communicating Health Care Information

Respondents again received the list of 6 electronic modes of communication and rated their preference for receiving information via each mode. They reported their preferences on a 5-point Likert scale that ranged from “Unwilling” to “Willing.” The same 6 options were repeated for 3 types of health information, including educational materials about cancer screening tests, tips for taking prescription medications, and recommendations on how to protect against infectious respiratory diseases including COVID-19.

#### Demographics and SDOH

The survey included items pertaining to respondents’ age, gender, race, ethnicity, residential location, income, educational achievement, and health status. Six demographic variables were used to describe respondents’ preferences by SDOH. The SDOH domain of social context was represented by age, gender, and race. Respondents reported their age in an open-ended question and chose gender from a provided list of options, including male, female, nonbinary or third gender, and others. Respondents selected their race from a multiple-choice question. The SDOH domain of neighborhood was identified by respondents' residential location by choosing one of the options indicating where they live: “large city,” “suburb near a large city,” “small city or town,” or “rural area.” For the SDOH domain of socioeconomic status, respondents reported their income by choosing one of 4 statements, “finding it very difficult on present income,” “finding it difficult on present income,” “getting by on present income,” and “living comfortably on present income.” For the SDOH domain of education, respondents provided their responses on a multiple choice item, where the responses ranged from “less than 8 years” to “postgraduate” education.

### Data Transformation and Analysis

#### Preferences for Modes of Communicating Health Care Information

To summarize the respondents’ preferences, we created dichotomous variables for each of the 6 modes of communication. Respondents were considered “willing” to use a specific mode of communication if they chose option 5 (“willing”) or 4 (“somewhat willing”). Respondents were considered “unwilling” to use a specific mode of communication if they chose any of the other options.

#### SDOH Domains

For analyses, we categorized age using the median age, resulting in 2 categories “less than 65 years” and “more than 65 years.” Gender was similarly considered in only 2 categories (female and male) because no respondent reported otherwise. For each of the other variables, we used resulting data distributions to determine categories. When evaluating differences by race, we included only Black and White respondents due to a small number of respondents in other categories (n=1). The location variable was split into rural and nonrural categories. Income was categorized into 2 groups. We included those who suggested it was “very difficult,” “difficult to live on their present income,” or “getting by on present income” in the category “low income.” The “high income” category included respondents who reported “living comfortably on present income.” The education domain included respondents with a college education who graduated with a bachelor's degree or higher and without college education reflecting those reporting less education.

#### Statistics

We report the number and percentage of respondents who were coded as “willing” to receive each mode of communication. We report means and SDs for continuous SDOH variables and frequencies and percentages for categorical ones. We used Pearson chi-square analysis for selected comparisons of respondents' willingness to receive electronic communication via specific modes. The difference in preferences was considered significant at *P*≤.05. This was exploratory formative research to establish differences among the older patient population by SDOH to inform future research. We did not conduct sample size calculations a priori, but estimated sample size based on methodological suggestions [32]. The comparison was performed if the difference between groups reached at least 10% and the group included more than 5 respondents. We excluded demographic missing data from each analysis using a casewise approach.

#### Missing Data

Respondents did not answer some questions about their preferences for electronic modes of communication, which resulted in 10 to 22 missing entries in the variables that measured modes of communication. The missing data were not random. We observed that respondents failed to provide answers mostly to the least preferred modes of communication. These patterns were consistent across different types of communication. Because of this, when we report the percentage of respondents who are willing to receive communication from a provider by each specific mode of communication (eg, telephone and patient portal), in the denominator, we use the sum of respondents who were willing to receive, unwilling to receive, or did not provide an answer to the item. In doing so, the number of participants remains the same across different modes of communication, which allows comparison and data interpretation. However, in the analyses that compared communication modes within each SDOH domain, we excluded missing values and compared respondents who were “willing” or “unwilling” to use a specific mode of communication. We report the number of participants, along with statistical summaries. The missing data for each mode of communication is reported in Multimedia Appendix 2.

## Results

In total, 133 completed surveys were returned. Of these, only 5 were completed online. The response rate was 27%. Table 1 presents sample demographic characteristics and other SDOH domains. Respondents’ age ranged from 47 to 78 years, with a mean of 64 (SD 8) years and a median of 65 years. There were more female (n=82, 63%) than male (n=48, 37%) respondents, respectively. Most of the respondents were White (n=106, 83%) or Black (n=20, 16%), only 1 respondent reported being Asian, and 6 respondents did not answer the question about their race. In total, 75 (58%) respondents reported having completed a college education and 54 (42%) did not have a college education. Approximately, half of the respondents (n=73, 57%) reported “living comfortably” with their current income. Respondents reported living in cities (n=15, 12%), suburban areas (n=37, 29%), towns (n=50, 39%), and rural areas (n=26, 20%).

**Figure 1:**
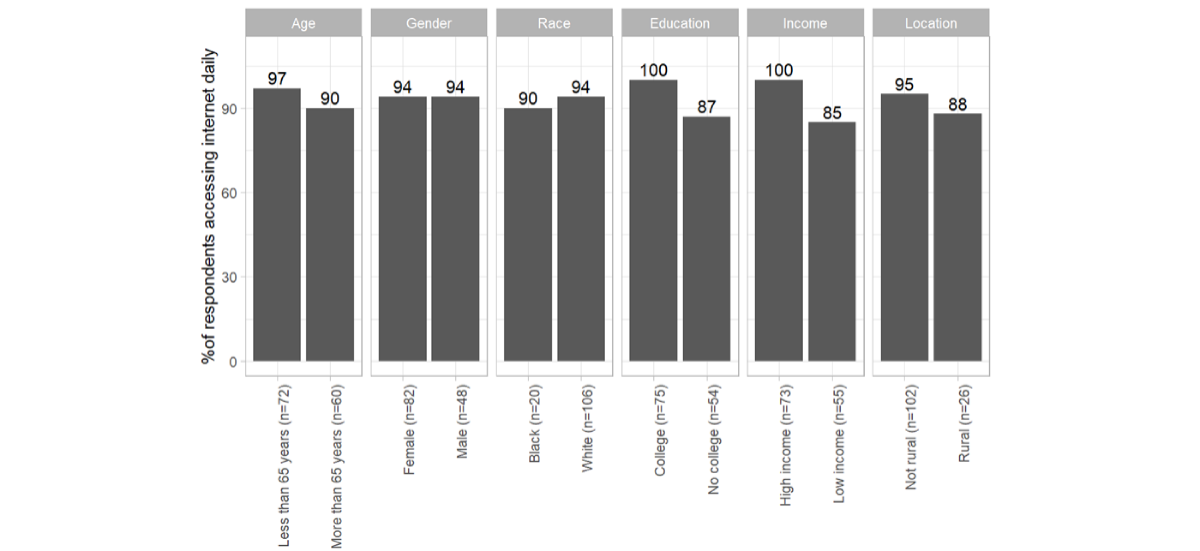
Access to the internet on a computer, tablet, or smartphone, daily.

Most respondents (n=125, 94%) had access to a computer, tablet, or smartphone, while only 3 (2%), indicated that they did not have access to any of the devices including a landline telephone. Access to electronic devices varied by social characteristics reported in Figure 1. The notable discrepancies are in percentages of respondents who have or have not obtained a college degree, among patients with different levels of income, and living in different locations, more details are reported in Multimedia Appendix 3.

The majority of respondents (n=112, 86%) indicated having a reliable internet connection at home. In total, 90 (69%) respondents reported using texting daily and 63 (54%) respondents reported viewing social media including Facebook, Twitter, Instagram, or YouTube daily. Respondents reported receiving health care information from their health care team using one or several modes of communication such as the patient portal (n=95, 71%), email (n=63, 47%), telephone calls (n=54, 41%), and SMS text messages (n=40, 30%). A more detailed report about current communications by social characteristics is presented in Multimedia Appendix 3.

**Figure 2:**
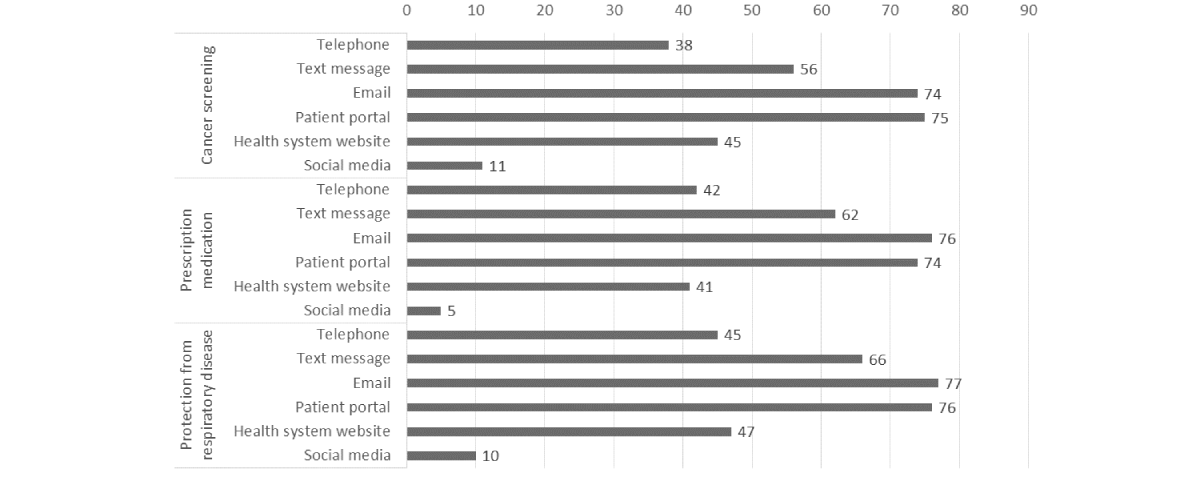
Average percentage of respondents' preferences by type of information and mode.

In our sample, the majority of respondents reported preferring to use the patient portal (n=100, 75%) and email (n=98, 74%), which was closely followed by SMS text messages (n=75, 56%) for receiving cancer screening information. Some respondents (n=60, 45%) preferred to look up information on the hospital website or receive a telephone call (n=50, 38%). The least preferable option was social media (n=14, 11%). The patterns for different types of communication (prescription medication and protecting against respiratory diseases) were similar with leading choices to be patient portals and emails, and the least preferred option was social media. The statistics are reported in Figure 2 and in Multimedia Appendix 2.

**Figure 3:**
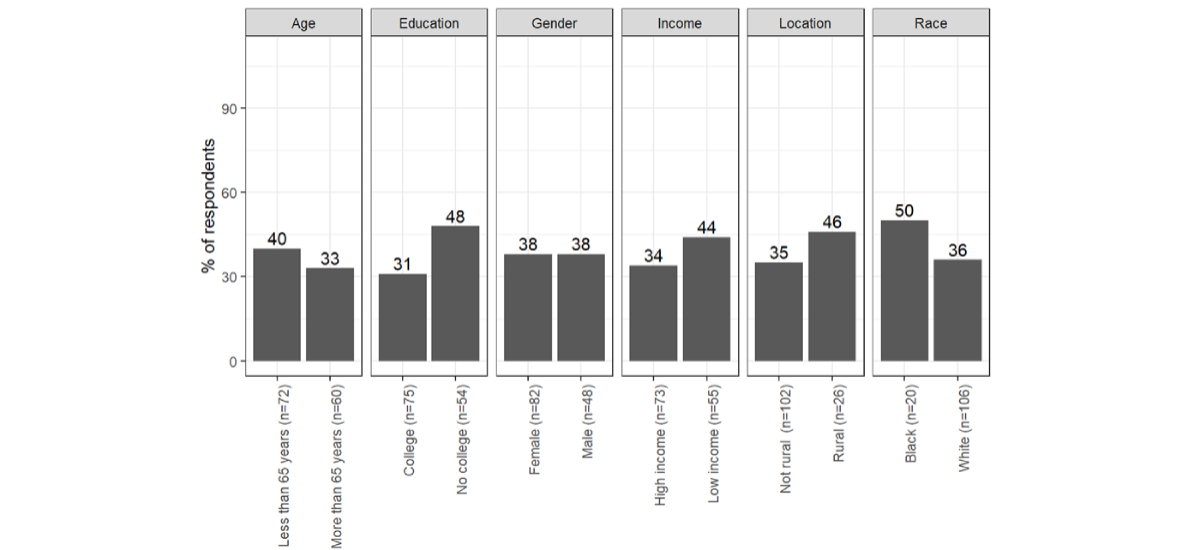
Percentage of respondents who prefer physicians to contact them via telephone calls: cancer screening information.

**Figure 4:**
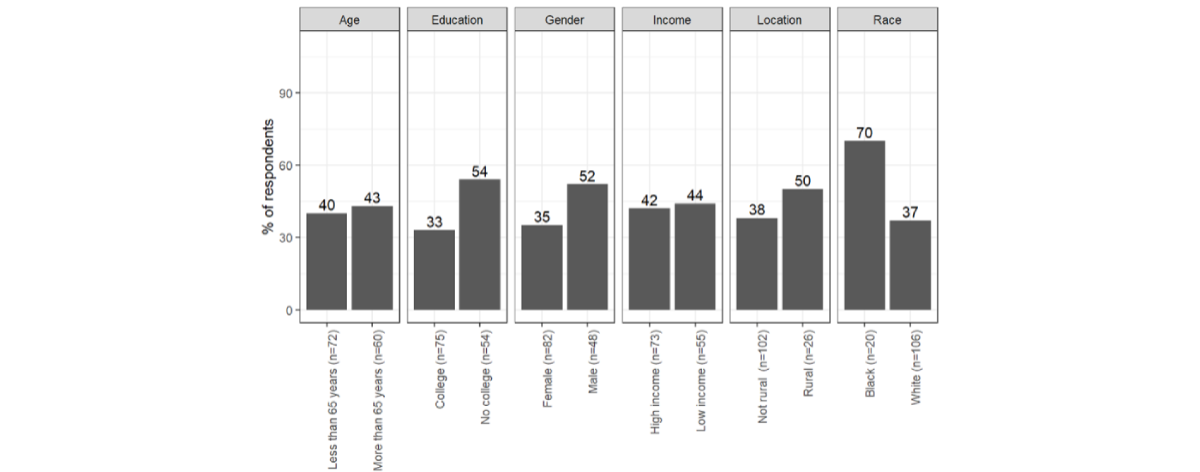
Percentage of respondents who prefer physicians to contact them via telephone calls: prescription medication.

**Figure 5:**
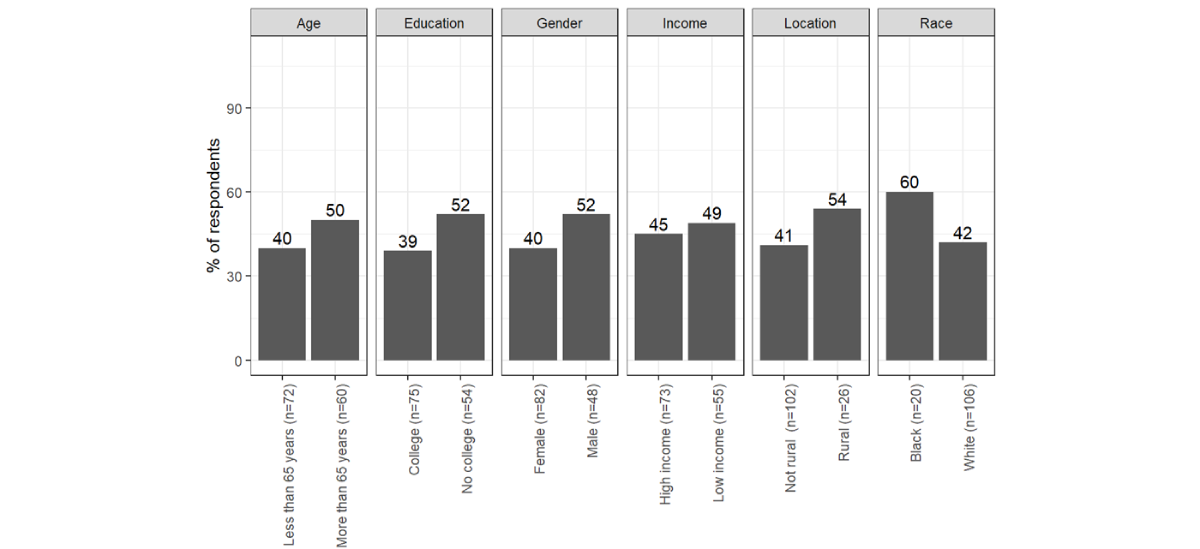
Percentage of respondents who prefer physicians to contact them via telephone calls: respiratory disease protection.

The most consistent differences between respondents with different social characteristics were concerning telephone calls and the patient portal. Communication preferences for a telephone call differed by race and education. Black respondents preferred telephone calls more than White respondents with regard to information about protection from respiratory disease (n=116, 60% vs 42%; *χ*^2^_115_=4.0; *P*=.046). Respondents who reported less education preferred telephone calls more than respondents with more formal education about information related to cancer screening (n=115, 48% vs 31%; *χ*^2^_114_=9.0; *P*<.01), prescription medication (n=120, 54% vs 33%, *χ*^2^_119_=8.7; *P*<.01), and protection against respiratory diseases (n=120, 52% vs 39%, *χ*^2^_119_=4.5; *P*=.04). Figures 3-5 illustrate the differences in social characteristics for telephone calls.

**Figure 6:**
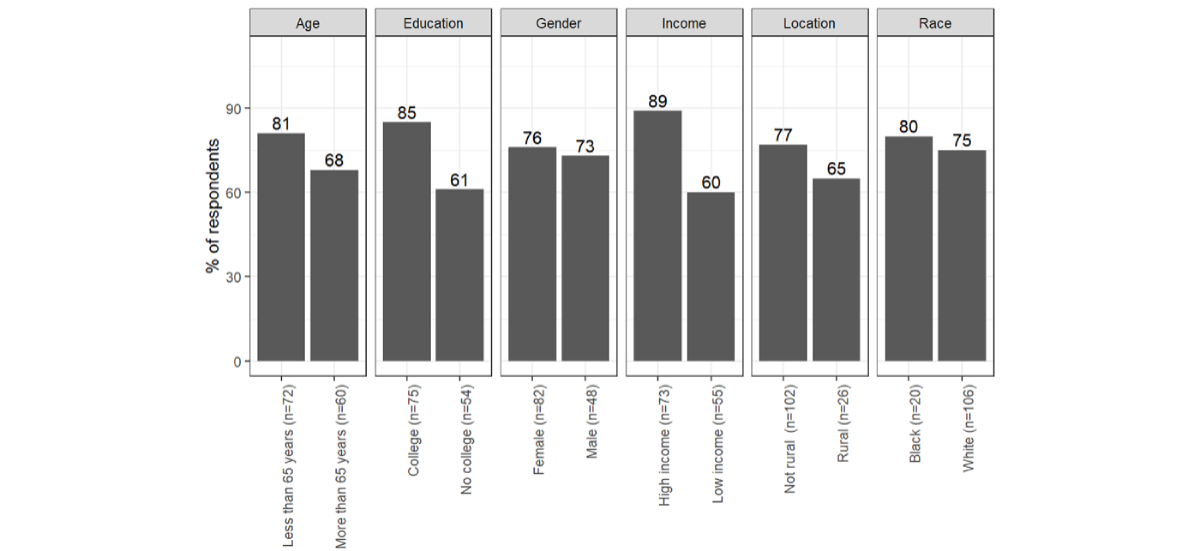
Percentage of respondents who prefer physicians to contact them via patient portals: cancer screening.

**Figure 7:**
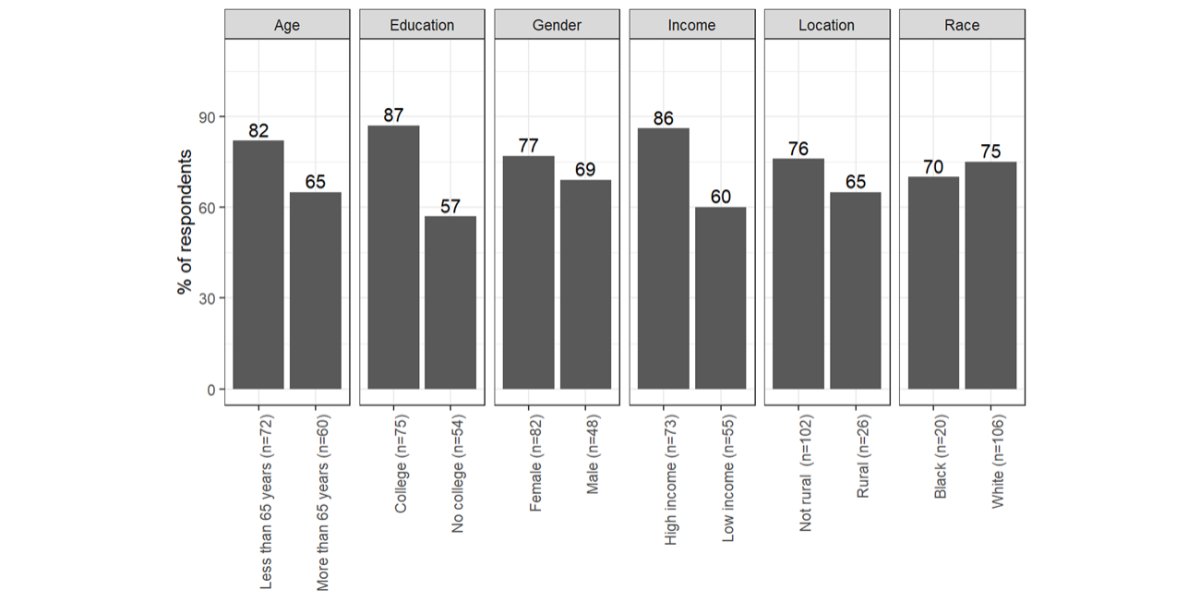
Percentage of respondents who prefer physicians to contact them via patient portals: prescription medication.

**Figure 8:**
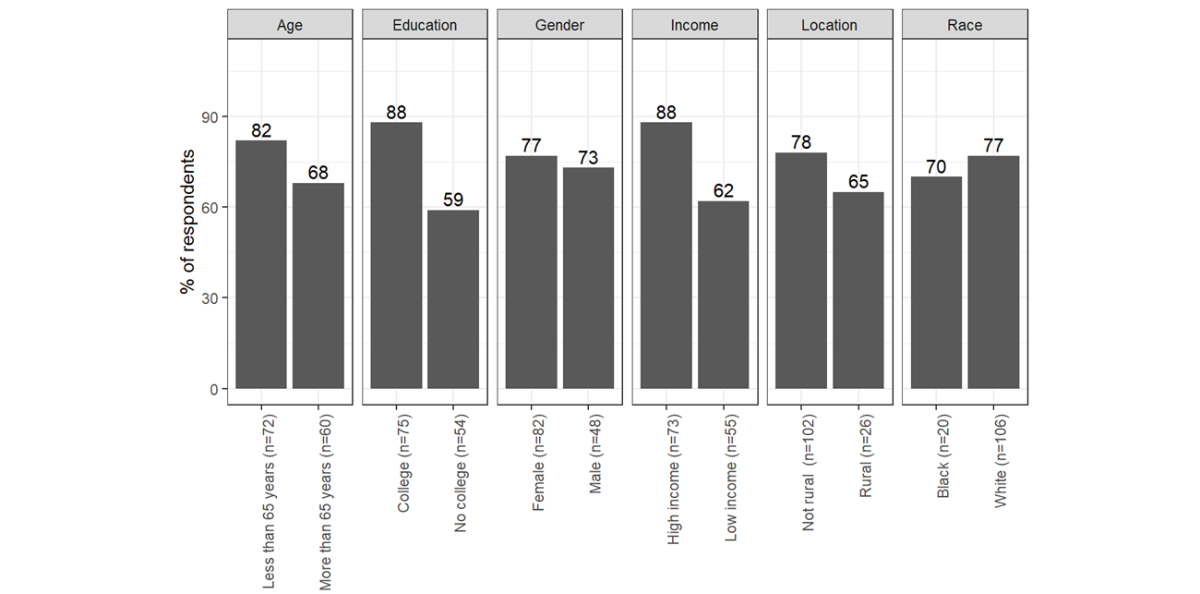
Percentage of respondents who prefer physicians to contact them via patient portals: respiratory disease protection.

The other consistent differences were in respondents’ preferences for using patient portals as reported in Figures 6-8. Respondents with a bachelor's degree or higher education preferred to receive information via patient portals more than respondents with less formal degrees with regard to cancer screening (n=118, 85% vs 61%; *χ*^2^_117_=3.9; *P*=.048), prescription medication (n=116, 87% vs 57%; *χ*^2^_115_=5.5; *P*=.02), and respiratory disease protection (n=116, 88% vs 59%, *χ*^2^_115_=5.3; *P*=.02). Respondents with a higher income were more willing to receive information via patient portals than respondents with a low income with regard to cancer screening (n=118, 89% vs 60%; *χ*^2^_117_=9.1; *P*<.01), prescription medication (n=116, 86% vs 60%; *χ*^2^_115_=8.7; *P*<.01), and respiratory disease protection (n=116, 88% vs 62%; *χ*^2^_115_=6.5; *P*=.01).

We also noted that respondents younger than 65 years preferred to receive information via email more than respondents who are older than 65 years (n=116, 82% vs 63%; *χ*^2^_115_=5.5; *P*=.02). This difference was only significant for communication about cancer screening.

We found gender differences in preferences for the hospital website. Male respondents preferred communication via the website more than female respondents with regard to information about cancer screening (n=112, 58% vs 38%, *χ*^2^_111_=4.3; *P*=.04) and prescription medication (n=110, 56% vs 33%; *χ*^2^_109_=5.3; *P*=.02).

**Table 1:** Sample characteristics.

Characteristic		Values (N=133)		Categories included in SDOH^a^ domains
Mean age (years), mean (SD)		64.19 (7.87)		More than 65 years old; less than or equal to 65 years old
**Age in deciles (years), n/N (%)**				
	50-59	40/129 (31)		
	60-69	50/129 (39)		
	≥70	39/129 (30)		
	Missing	4		
**Gender, n/N (%)**				Female and male
	Female	82/130 (63)		
	Male	48/130 (37)		
	Missing	3		
**Race, n/N (%)**				Black, White
	Asian	1/127 (0.8)		
	Black	20/127 (16)		
	White	106/127 (83)		
	Missing	6		
**Ethnicity, n/N (%)**				Not included
	Hispanic	1/112 (0.9)		
	Missing	21		
**Location of residence, n/N (%)**				Rural—a rural area; nonrural—all other nonmissing responses
	Large city	15/128 (12)		
	Suburb near a large city	37/128 (29)		
	Small city or town	50/128 (39)		
	Rural area	26/128 (20)		
	Missing	5		
**Education, n/N (%)**				College education—college graduate and postgraduate; No college education—all other nonmissing responses
	Less than high school	7/129 (5.4)		
	High school	13/129 (10)		
	Some college	34/129 (26)		
	College graduate	39/129 (30)		
	Postgraduate	36/129 (28)		
	Missing	4		
**Income comfort, n/N (%)**				High income—living comfortably on the present income; low income—all other nonmissing responses
	Living comfortably	73/128 (57)		
	Getting by	43/128 (34)		
	Finding it difficult	7/128 (5.5)		
	Finding it very difficult	5/128 (3.9)		
	Missing	5		

^a^SDOH: social determinants of health.

## Discussion

### Principal Findings

In a cross-sectional study of primary care patients aged 47-78 years, we found the majority (92%) reported using electronic devices and accessing the internet daily. This percentage is somewhat higher compared to the results reported by the Health Information National Survey, which identified that 77% of the US population access the internet daily via mobile devices [33]. Consistently, we found that the majority of patients prefer to receive health information using the internet via patient portals and emails. These results would be relevant to populations, in which individuals are similar to the respondents in our sample, which include mostly White respondents who lived in a suburb or town area and reported being comfortable with their income.

More importantly, we conducted an analysis exploring the preferences of respondents by SDOH. Patients suggest that SDOH are the key contributing factors to the growing digital divide in health care communication [34]. Our results revealed consistent differences in patients' preferences with regard to telephone and patient portal communication. Income and education were the factors that divided participants’ opinions the most. Respondents’ willingness to receive health messages via patient portals was substantially lower among people with low income and no college education. Consistently, respondents with no college degree preferred telephone calls more than those with college degrees. Another difference in preferences was observed between Black and White participants. Black participants chose telephone calls more frequently than White participants. The difference was consistent for every type of communication and reached statistical significance for the communication about protection from respiratory diseases. Further research is needed to understand the reasons underlying why some populations were less willing to communicate via patient portals and more willing to receive telephone calls.

Our results identify important differences in communication preferences among populations with different SDOH. As such, our findings can inform how policies and practices regarding modes of communication can be used to ensure that critical health information and services are accessible to all in a timely manner. For example, behavioral information programs that target chronic diseases or cancer screening tests may consider using patient portals as a primary mode of communication when wanting to reach people who do not face financial insecurity and who have a college education. However, the same programs should consider using telephone calls to ensure reaching patients who have less than a college education or financial insecurity as not doing so could result in structural biases and ultimately, structural care inequities. As such, our findings contribute important information to those developing guidelines for electronic communications, which to date, lack evidence and provide minimum recommendations on how to use electronic tools with patients effectively [35].

In contrast with previous research [36] suggesting age as a critical barrier to the adoption of electronic communications, we observed that the majority of our respondents preferred receiving health information from their doctor’s office via patient portals or email. Interestingly, when we divided participants into 2 groups with a median of 65 years, we did not observe significant differences in preferences between the older and younger group. The only difference in preferences for emails was observed with regard to cancer screening information. Older participants preferred to communicate via email less than younger participants. Despite this difference, our findings are rather consistent with the research, showing that electronic communication can be effective for [37] and liked by [38] older populations. Therefore, age alone should not be a factor based on which electronic modes of communication are excluded, rather patients’ preferences, regardless of age should be assessed to ensure a match.

Comparing our work with the previous research, we suggest that our results might be reflective of a shift in preferences over time. A study conducted in 2012 among a general US population [16] showed that 75% of respondents at that time preferred to receive a telephone call from a provider, 49% wanted to communicate via email, and only 13% chose SMS text messaging. The relatively rapid changes in the availability and acceptability of electronic communication led to patients becoming more familiar with and accustomed to electronic services. Rapid changes in patient preferences require health care organizations not only to adapt to the needs of different populations but also to assess their needs routinely and continue adaptation providing information to patients in a timely manner. Assessing and accommodating patients’ preferences for electronic (and other) modes of communication is now a critical component of health care delivery as care continues to transition from face-to-face encounters within traditional doctor’s offices to more varied settings.

### Limitations

A limitation of our study is a low (27%) response rate. While this response rate warrants caution when generalizing findings, it is consistent with recently observed response rates in similar contexts [39]. Participants for the study were recruited via random sampling, and while we attempted to recruit a representative sample of participants, our study sample may differ from the underlying population in both measured and unmeasured ways.

Our sample was drawn randomly from an eligible patient population recently presenting to primary care. The underrepresentation of Black adults relative to those receiving primary care at the organization is likely due to multiple social factors [40]. The same may be true of those with relatively limited income. Our results need to be interpreted in light of such limitations. For instance, we observe consistent differences in Black and non-White patients’ preferences for phone communication. Although these differences did not reach a statistically significant level, results, nonetheless, may be indicative of true differences in preferences between those populations. We also compared the preferences of participants who reported lower income, including those who suggested “getting by on present income” with respondents who reported “living comfortably on present income.” The observed significant differences suggest that not only people who experience substantial difficulty living with their present income need support to adopt patient portals but also people who manage to meet their needs on their present income.

Data missingness was another challenge. Within some variables, such as preferences for communicating via social media, we observed missing data in 22% of cases. Notably, the pattern of missingness closely resembled the distribution of patients’ preferences, namely the least preferred modes of communication had the most instances of missing data. We might speculate that missingness was associated with respondents' unwillingness to use a specific means of communication. However, to ensure the robustness of our results, we excluded missing data in the analysis that included statistical comparisons for each of these variables.

Our results should be generalized in light of the measured demographic characteristics. Caution should be exercised when attempting to extend the conclusions beyond the present sample, especially with respect to individuals under the age of 45 years or those who belong to demographic categories that were not represented in our sample.

### Conclusions

We found that the majority of the adult primary care patients recruited for the study strongly preferred electronic communications, such as patient portals and email. We also noted substantial differences in respondents’ preferences across populations defined by SDOH. Our findings suggest that to reach socioeconomically diverse primary care patient populations with critical health information, health care providers need to add telephone calls, especially when communicating with people who have low income and no college education. Further research should focus on identifying interventions that will support the adoption of electronic communications, particularly, patient portals among those populations.

## Appendix

Multimedia Appendix 1 Survey: relevant items.

Multimedia Appendix 2 Respondents preferences and missing data.

Multimedia Appendix 3 Respondents who have access to electronic communication.

## Abbreviations

SDOH: social determinants of health

## Data Availability

Data are available from the authors upon request.
