# Supplementary material for: Preferences for Electronic Modes of Communication Among Older Primary Care Patients: Cross-sectional Survey
Source: JMIR Form Res. 2023 May 24;7:e40709. doi: 10.2196/40709 (PMC10248769; doi:10.2196/40709)
Supplement: Multimedia Appendix 1 [file formative_v7i1e40709_app1.docx]

**Appendix 1**

1. Do you own or have access to any of the following devices? Please mark all that apply.


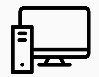


- Computer (for example, a desktop or laptop)


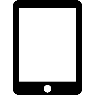


- Tablet (for example, an iPad or Surface)


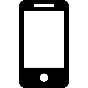


- Smartphone (for example, an iPhone or Android)


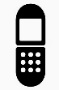


- Basic cell phone (for example, a flip phone)


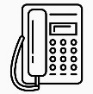


- Landline phone
- I do not own or have access to any of the devices listed 🡪 Skip to Q18.

1. From among the list below, please indicate how you currently receive educational health information from your doctor’s office. Please mark all that apply.

- Phone call from an automated system
- Text message
- Email
- Patient portal message (e.g., My Chart)
- YouTube
- Facebook
- Instagram
- Twitter
- [Name] Health Care’s website

1. If your doctor’s office were to provide educational information on cancer screening tests, how willing would you be to receive that information via the following methods?

|  | Unwilling | Somewhat unwilling | Neither willing or unwilling | Somewhat willing | Willing |
| --- | --- | --- | --- | --- | --- |
| 1. Phone call from an automated system |  |  |  |  |  |
| 1. Text message |  |  |  |  |  |
| 1. Email |  |  |  |  |  |
| 1. Patient portal message (e.g., My Chart) |  |  |  |  |  |
| 1. YouTube |  |  |  |  |  |
| 1. Facebook |  |  |  |  |  |
| 1. Instagram |  |  |  |  |  |
| 1. Twitter |  |  |  |  |  |
| 1. [Name] Health Care’s Website |  |  |  |  |  |

1. If your doctor’s office were to provide tips for taking prescription medications, how willing would you be to receive those tips via the following methods?

|  | Unwilling | Somewhat unwilling | Neither willing or unwilling | Somewhat willing | Willing |
| --- | --- | --- | --- | --- | --- |
| 1. Phone call from an automated system |  |  |  |  |  |
| 1. Text message |  |  |  |  |  |
| 1. Email |  |  |  |  |  |
| 1. Patient portal message (e.g., My Chart) |  |  |  |  |  |
| 1. YouTube |  |  |  |  |  |
| 1. Facebook |  |  |  |  |  |
| 1. Instagram |  |  |  |  |  |
| 1. Twitter |  |  |  |  |  |
| 1. [Name] Health Care’s Website |  |  |  |  |  |

1. If your doctor’s office were to provide information on how to protect yourself from infectious respiratory diseases (For example, flu or COVID-19), how willing would you be to receive that information via the following methods?

|  | Unwilling | Somewhat unwilling | Neither willing or unwilling | Somewhat willing | Willing |
| --- | --- | --- | --- | --- | --- |
| 1. Phone call from an automated system |  |  |  |  |  |
| 1. Text message |  |  |  |  |  |
| 1. Email |  |  |  |  |  |
| 1. Patient portal message (e.g., My Chart) |  |  |  |  |  |
| 1. YouTube |  |  |  |  |  |
| 1. Facebook |  |  |  |  |  |
| 1. Instagram |  |  |  |  |  |
| 1. Twitter |  |  |  |  |  |
| 1. [Name] Health Care’s Website |  |  |  |  |  |
